# Simplified intravoxel incoherent motion diffusion-weighted MRI of liver lesions: feasibility of combined two-colour index maps

**DOI:** 10.1186/s41747-021-00233-1

**Published:** 2021-08-09

**Authors:** Petra Mürtz, Narine Mesropyan, Alois M. Sprinkart, Wolfgang Block, Julian A. Luetkens, Ulrike Attenberger, Claus C. Pieper

**Affiliations:** Department of Diagnostic and Interventional Radiology, University Hospital Bonn, Venusberg-Campus 1, D-53127 Bonn, Germany

**Keywords:** Diffusion magnetic resonance imaging, Feasibility studies, Focal nodular hyperplasias, Hemangioma, Liver neoplasms

## Abstract

**Background:**

To evaluate the feasibility of two-colour index maps containing combined diffusion and perfusion information from simplified intravoxel incoherent motion (IVIM) for liver lesion malignancy assessment.

**Methods:**

Diffusion-weighted data from a respiratory-gated 1.5-T magnetic resonance sequence were analysed in 109 patients with liver lesions. With three *b* values (0, 50, 800 s/mm^2^) estimated diffusion coefficient D′, perfusion fraction f′, and apparent diffusion coefficient (ADC) maps were calculated and analysed for regions of interest (ROIs). D′ and f′ cutoff values were determined by differentiating haemangiomas from other lesions and focal nodular hyperplasias from other lesions, respectively. Combined I_Df_ index maps were generated with a voxel value set to 100, if both D′ and f′ voxel values were lower than their cutoff values (1,529.4 × 10^-6^ mm^2^/s and 114.4 × 10^-3^, respectively), otherwise to 0. Moreover, I_ADC_ index maps were generated from ADC cutoff value (1,338.5 × 10^-6^ mm^2^/s) obtained by differentiating benign from malignant lesions. Discriminatory power was assessed for both I_Df_ and I_ADC_. Index maps were displayed as two-colour overlays to *b*-800 images and visually assessed within the translucent hyperintense areas.

**Results:**

For I_Df_, the same diagnostic accuracy was achieved as for the combined use of parameters D′ and f′ (93.6%). Compared to I_ADC_, I_Df_ showed a higher diagnostic accuracy. Visual judgment of I_Df_ yielded an accuracy (95.4%) similar to that of quantitative analysis (93.6%).

**Conclusion:**

Voxel-wise combined two-colour index maps I_Df_ provide similar diagnostic accuracy as ROI-based combination of estimated IVIM parameters D′ and f′ and are suitable for visual assessment of liver lesion malignancy.

## Key points

- Index map I_Df_ can replace the combined use of D and f parameters.
- Two-colour *b*-800 overlay I_Df_ enables a visual assessment of liver lesion malignancy.
- Visual judgment and quantitative analysis of I_Df_ showed comparable diagnostic accuracy.

## Background

Diffusion-weighted imaging (DWI) is an important magnetic resonance imaging (MRI) technique for detection and differentiation of liver lesions not needing contrast agent administration and should be implemented in standard liver examination in routine clinical practice [1].

While DWI acquired with a low *b* value (“black blood” images) provides high sensitivity for lesion detection [2, 3], the apparent diffusion coefficient (ADC) determined from at least two *b* values between 0 and 500–1000 s/mm^2^ is usually used for lesion characterisation [4, 5]. The intravoxel incoherent motion (IVIM) concept enables the separation of diffusion and perfusion effects on the DWI signal by assuming a biexponential behaviour of signal intensity [6–8]. The true diffusion coefficient D, the pseudodiffusion coefficient D*, and the perfusion fraction f, reflecting the relative contribution of perfusion to the DWI signal, are often determined by fit algorithms [9]. These require a high number of *b* values and thus relatively long acquisition times. Limited data quality due to signal variations caused by respiratory and cardiac motion and due to low signal-to-noise ratio may lead to unstable fitting results, measurement errors, and poor reproducibility [10–13]. Improved stability and lower acquisition times can be achieved by so-called “simplified IVIM”, which uses explicit computation of IVIM numerically stable parameter estimations in combination with a small number of *b* values. Simplified IVIM turned out to be valuable for liver lesion characterisation and assessment of therapy in clinical routine [3, 14–20].

For lesion assessment, voxel-wise evaluation and the creation of parameter maps are important. Still somewhat inconvenient for clinical use is the quantitative analysis of regions of interests (ROI) in the IVIM parameter maps. The use of colour-coded maps [21, 22] as overlay over b_0_ DWI images [14–16, 23] enables visual lesion assessment. For the assessment of malignancy, knowledge of the cutoff values of each IVIM parameter is necessary. From ischemic stroke diagnostic using computed tomography perfusion, the use of two-colour index maps is known allowing a rapid and easy image interpretation [24, 25]. Suitable two-colour index maps obtained from IVIM parameters could allow a rapid and easy image interpretation with respect to malignancy.

The purpose of this study was to create and evaluate two-colour index maps, which combines diffusion and perfusion information obtained by simplified IVIM for convenient visual assessment of liver lesion malignancy.

## Methods

### Study cohort

This retrospective study was approved by the local institutional review board of the University Hospital Bonn, Germany, with waiver for written informed patient consent. Data of 1,721 consecutive examinations (from February 2013 to September 2016) of patients, who received a 4 *b* value DWI sequence at 1.5 T, were reviewed. Data of 1350 examinations were not used because the patients had no liver lesions, only cysts, or lesions < 1 cm, or because it was not the first examination in the study time frame, so that data of 371 different patients with at least one focal liver lesion ≥ 1 cm other than cysts were included. Of these 371 patients, 262 (70.6%) were excluded due to lack of a definitive diagnosis based on histology or typical imaging characteristics (*n* = 46), local treatment of the liver (*n* = 143), insufficient image quality caused by motion artifacts (*n* = 27) or pixel misalignments (*n* = 5), unfavourable lesion location as close to prior biopsy or drainage tracts or at the edge of the liver (*n* = 6), partial volume of an adjacent slice (*n* = 10), or difficulties to identify the lesions on DWI (*n* = 5). In the presence of a combination of (non-cystic) benign lesions and malignant disease, patients were excluded because malignant disease may affect the appearance of benign liver lesions, *e.g.*, due to thrombosis (*n* = 20). Finally, data of 109 patients were analysed (Table 1).

**Table. 1:** Group composition and demographic data of included subjects

Liver pathologies	Total number of patients	Number of males	Age range (years)
Hepatocellular carcinoma	32	20	55–87
Cholangiocellular carcinoma	8	4	57–85
Metastases from colorectal cancer	22	17	47–87
Metastases from breast cancer	12	0	48–70
Haemangioma	23	12	34–84
Focal nodular hyperplasia	12	1	14–54
Total	109	54	14–87

These patients had already been examined in an upcoming study by Mesropyan et al and in a previous study [15], where basic investigations concerning simplified IVIM for liver lesion characterisation [15] and different ROI placement and analysis methods had been performed in an upcoming study by Mesropyan et al. In the present study, the data were used to evaluate two-colour index maps constructed with the help of IVIM parameter analysis results.

Cholangiocellular carcinomas (CCCs) were histologically proven. Hepatocellular carcinomas (HCCs) were either histologically proven or diagnosed according to the American Association for the Study for Liver Disease MRI criteria [26]. Diagnosis of metastasis was histologically proven or based on typical imaging features in combination with histologically proven primary cancer. Diagnosis of focal nodular hyperplasia (FNH) or haemangioma was established based on typical radiological findings on contrast-enhanced MRI and was confirmed by at least one follow-up examination.

### Magnetic resonance imaging

Imaging was performed on a clinical whole-body 1.5-T MRI system (Ingenia, Philips Healthcare, Eindhoven, The Netherlands) equipped with powerful gradient system (45 mT/m maximum amplitude, 200 T/m/s maximum slew rate) and 32-channel abdominal coil with digital interface for signal reception. DWI with a respiratory-triggered single-shot spin-echo echo-planar imaging variant (Table 2) with four *b* values (0, 50, 250, 800 s/mm^2^) was applied before contrast agent administration. Isotropic diffusion-weighted images were reconstructed by from the images with diffusion-sensitised gradients in three orthogonal directions on the MRI system.

**Table. 2:** Technical parameters of the diffusion-weighted imaging (DWI) sequence

Name	Value
Field of view (right-left × anterior-posterior)/orientation	380 × 326 mm/transversal
Slice number/thickness/gap	30/7.0 mm/0.7 mm
Matrix/resolution	112 × 94/3.4 × 3.5 mm
Echo time	63 ms
Repetition time	1 respiratory cycle
Imaging time per respiration	1,600 ms
Echo-planar imaging/half-Fourier/SENSE factor	51/0.6/2
Diffusion gradients	3 orthogonal directions
*b* values (number of averages per direction)	0, 50, and 250 s/mm^2^ (2); 800 s/mm^2^ (4)
Fat suppression methods	Spectral presaturation by inversion recovery, SPIR
Water-fat shift/bandwidth	9.2 pixel/23.6 Hz
Bandwidth in echo-planar imaging frequency direction	1,437.9 Hz
Acquisition time	Around 4 min (2:42 min:s without gating)

*SENSE* Parallel imaging with sensitivity encoding

### Postprocessing

IVIM parameters D and f as well as conventional ADC were calculated voxel-wise from *b* = 0, 50, and 800 s/mm^2^ by using the following approximations:

1 $$ {D}^{\prime }= ADC\left(\mathrm{50,800}\right)=\frac{\ln \left(S\left({b}_{50}\right)\right)-\ln \left(S\left({b}_{800}\right)\right)}{b_{800}-{b}_{50}} $$

2 $$ {f}^{\prime }=f\left(\mathrm{0,50,800}\right)=1-\frac{S\left({b}_{50}\right)}{S\left({b}_0\right)}\bullet {\mathit{\exp}}^{{\mathrm{D}}^{\prime}\bullet {b}_{50}} $$

3 $$ ADC= ADC\left(\mathrm{0,800}\right)=\frac{\ln \left(S\left({b}_0\right)\right)-\ln \left(S\left({b}_{800}\right)\right)}{b_{800}-{b}_0} $$

Parameter maps and two-colour index maps (see below) were calculated offline using custom written software in MATLAB (MathWorks, Natick, Massachusetts, USA).

### Image analysis

Image analysis by ROIs was performed by a radiologist (N.M.) with 3 years of experience and checked by a radiologist (C.C.P.) with 10 years of experience in abdominal imaging and a physicist (P.M.) with more than 20 years of experience in DWI. All were blinded to clinical information. One reference lesion per lesion type was analysed. A two-dimensional ROI was placed centrally in each lesion on a single representative slice. This slice was largely unaffected by motion and susceptibility artifacts and pixel misalignments and not at the rim of the lesion to avoid partial volume effects. ROIs were drawn as large as possible using DWI with the highest contrast between lesion and normal tissue. Central necrosis, cystic components, and scars as found by hyperintensities on b0 images and/or hypointensities on *b*-800 images were excluded in an upcoming study by Mesropyan et al. After visually cross-checking for pixel misalignments between images with different b values, the ROI was analysed in the related parameter maps ADC, D′, and f′ and saved for later use (see below).

### Construction of two-colour index maps

Two-colour index maps I_ADC_, I_D_, and I_f_ were constructed from suitable cutoff values for ADC, D′, and f′, respectively. The cutoff values were determined as previously introduced [14, 15]: the ADC cutoff value was determined by receiver operating characteristic (ROC) analysis of malignant and benign lesion groups; for the combined use of D′ and f′, the D’ cutoff value was determined by differentiation between haemangiomas and all other lesions and the f′ cutoff value by differentiation between FNHs and all other lesions. Motivated by the high diffusion coefficient of haemangiomas [14, 15, 27, 28] and the high perfusion fraction of FNHs [14, 15, 29], lesions were assigned as malignant if ROI-wise mean values of D′ and f′ were both below their cutoff values, and otherwise as benign. In the index maps, a voxel value was set to 100 if the corresponding parameter voxel value was lower than the determined cutoff value; otherwise, the voxel value was set to 0. By combining I_D_ and I_f_, the index map I_Df_ was generated. For I_Df_, a voxel value was set to 100 if the corresponding voxel values of I_D_ and I_f_ were both 100; otherwise, the voxel value was set to 0. Voxel values 0 and 100 were displayed in green and red, respectively, indicating benign and malignant structures. These index maps were displayed as overlay over the DWI *b*-800 images.

### Evaluation of the two-colour index maps

First, to ensure that the voxel-wise consideration of the cutoff values does not worsen diagnostic performance compared to ROI-wise, for I_ADC_, I_D_, and I_f_, the same ROC analysis was performed as for the related original parameter (see the "Construction of two-colour index maps" section). AUC values were compared pairwise. Second, to compare the diagnostic performance of ADC, D′, and f′ as well as of I_Df_, I_ADC_, and ADC, all maps were quantitatively analysed using ROIs (see the “Image analysis” section). ROC analyses of the benign and malignant lesion groups were then performed. AUC values were compared with each other.

Third, the I_ADC_ and I_Df_ index maps were evaluated visually by one investigator (P.M.). The visual assessment was restricted to areas of translucent hyperintensity from DWI *b*-800 images, whereby necrosis, cystic components, and scars identified as hyperintense areas on *b*-0 images and/or hypointense areas on *b*-800 images were excluded. A four-point scale was used, as follows: (1) definitely malignant, if the red voxels dominated definitely; (2) probably malignant, if red voxels dominated only slightly; (3) probably benign, if green voxels dominated only slightly; and (4) definitely benign, if green voxels dominated definitely.

The accuracy of I_ADC_ and I_Df_ for lesion differentiation by visual assessment was determined and compared with each other and with ADC.

The assessment was repeated after 4 months by the same investigator (P.M.) and by a second independent investigator (C.C.P.).

### Statistical analysis

Statistically significant differences (*p* < 0.05) between groups (independent samples) were tested in SPSS (version 24.0, IBM, Armonk, New York , USA) by using Student *t* test or non-parametric Mann–Whitney *U* test, depending on whether the data were normally distributed or not. In order to differentiate between two groups, ROC analysis was performed using pROC package in R (version 1.17.0.1, open source package, accessible at http://expasy.org/tools/pROC/ under the GNU General Public License) [30]. Youden’s index was used to determine the optimal cutoff value of the ROC curve providing the highest combination of sensitivity and specificity. DeLong method was used to compare the area under the curve (AUC) of dependent and independent ROC curves [31]. The intraclass correlation coefficients (ICCs) were calculated for the visual assessment results of the same investigators (ICC_intra_) and of the two different investigators (ICC_inter_).

## Results

**Fig. 1:**
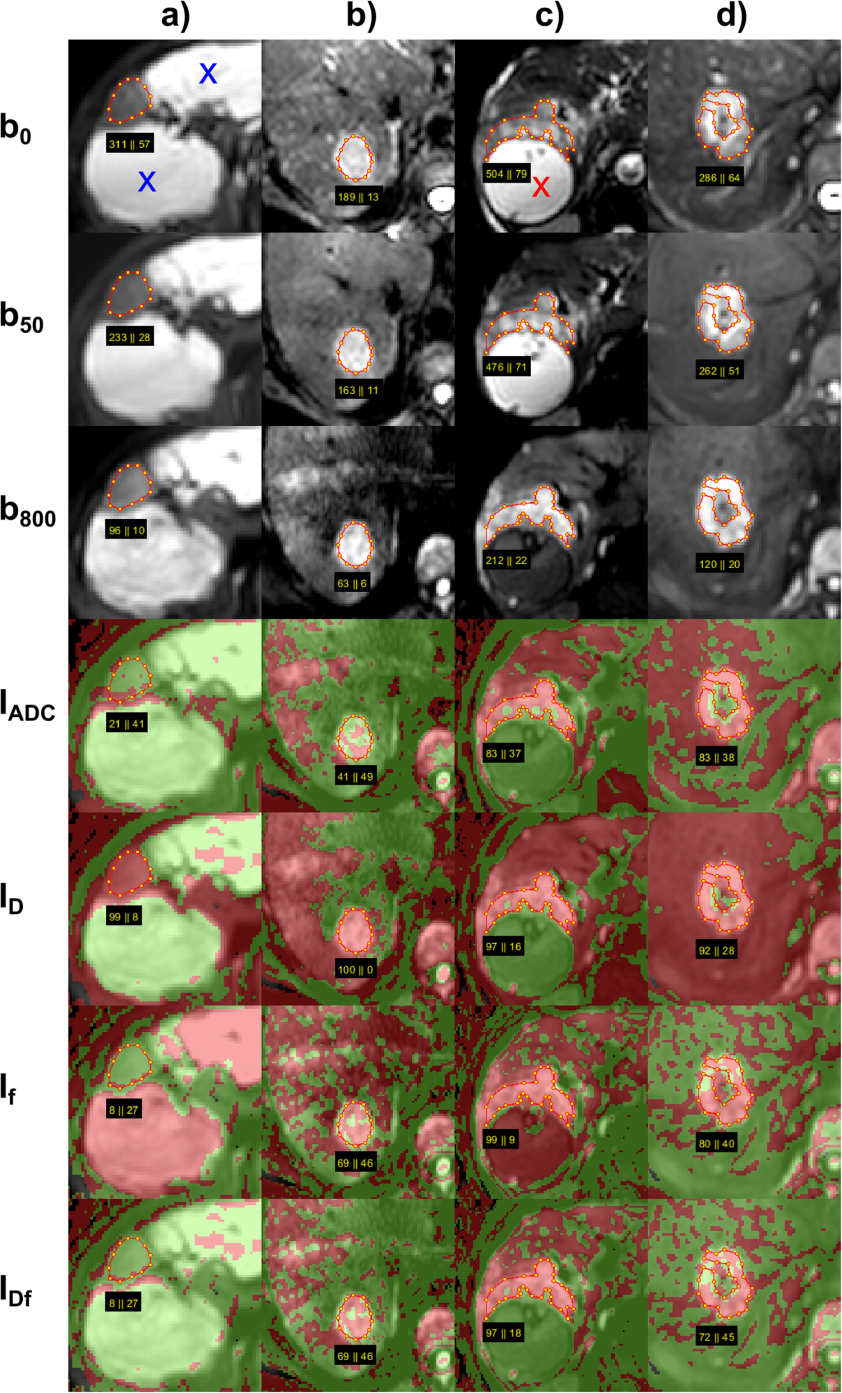
Typical examples of two-colour index maps. Combined index maps I_Df_ are given in comparison to index maps I_ADC_ together with I_D_ and I_f_, overlaid to *b*-800 images, for (**a**) FNH together with two haemangiomas (blue x), (**b**) HCC, (**c**) CCC together with bilioma (red x), and (**d**) metastasis from colorectal carcinoma (CRC). The FNH reveals almost everywhere voxels with perfusion fraction above cutoff (I_f_ green) so that I_Df_ shows clear benignity despite diffusion coefficient below cutoff (I_D_ red), I_ADC_ showed slightly less green voxels compared to I_Df_ (79% *versus* 92%). The haemangiomas shows almost everywhere voxels with diffusion coefficient above cutoff (I_D_ green), so that I_Df_ shows clear benignity despite the low perfusion fraction (I_f_ red), the same is valid for I_ADC_. The HCC shows area-wide diffusion coefficient below cutoff (red I_D_), and mainly perfusion fraction below cutoff with heterogeneous distribution (I_f_ scattered red) and thus also I_Df_ showing clear malignancy, I_ADC_ shows less red voxels compared to I_Df_ (41% *versus* 69%) showing benignity, the visual assessment was “probably” benign. The CCC appears on all maps mainly red showing clear malignancy on I_Df_ and I_ADC_ maps. The bilioma looks identical to the haemangiomas. The CRC reveals mainly red voxels within the selected region of interest, which excluded hypointense region on *b*-800 image (necrosis). *CCC* Cholangiocellular carcinoma, *CRC* Metastasis from colorectal carcinoma, *FNH* Focal nodular hyperplasia, *HCC* Hepatocellular carcinoma

**Fig. 2:**
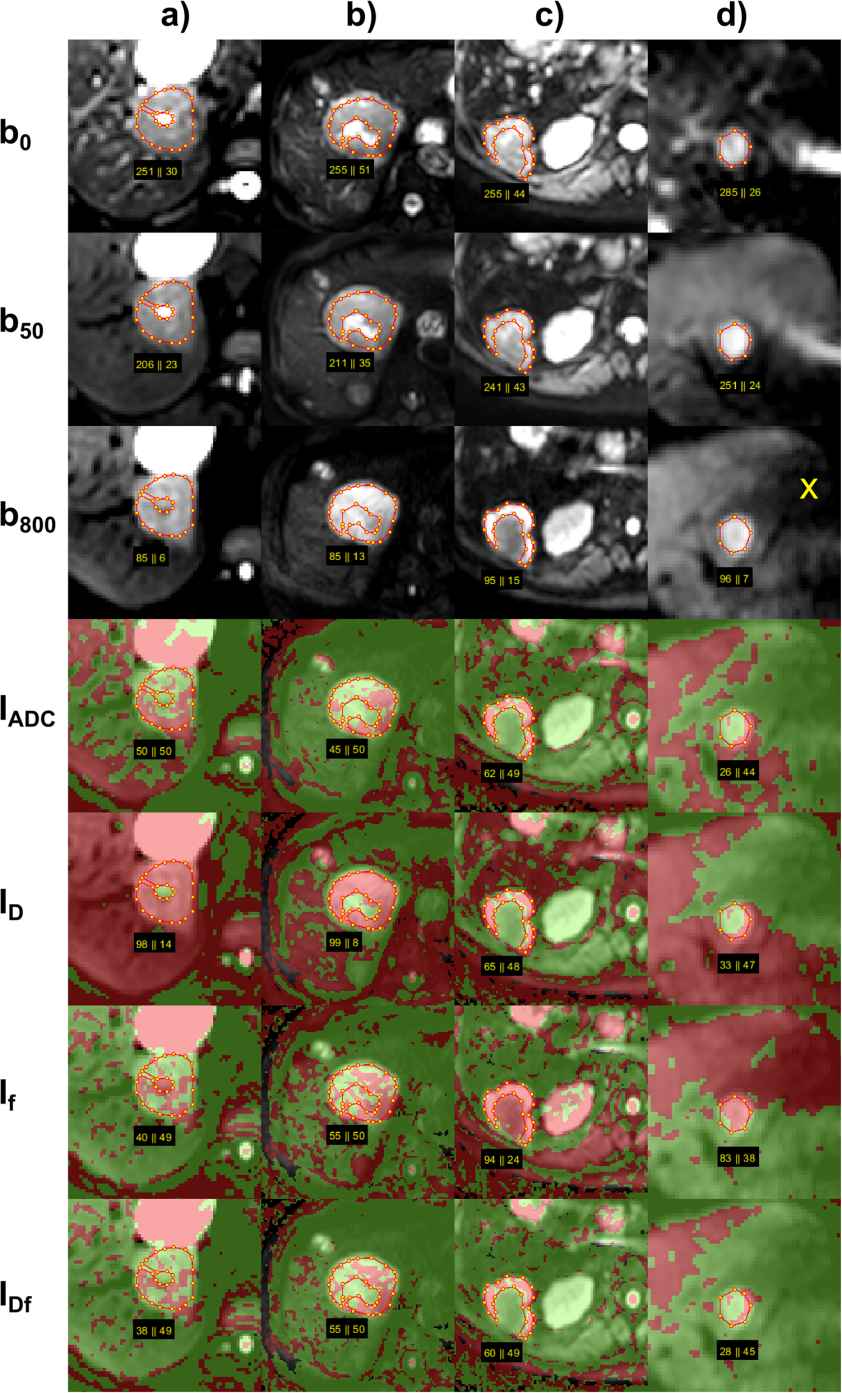
Further examples of combined two-colour index maps. Combined index maps I_Df_ are given in comparison to index maps I_ADC_ together with I_D_ and I_f_, overlaid to *b*-800 images, for (**a**) FNH, (**b**) HCC, (**c**) metastasis of breast cancer, and (**d**) HCC. The FNH with central scar (**a**) and the HCC with central necrosis (**b**) show similar behaviour on all index maps with nearly all voxels being red on I_D_ and about half of the voxels being red with scattered distribution on I_f_ and also on I_ADC_ and I_Df_. On I_ADC_, visual assessment of FNH is “probable malignant” and of HCC “probably benign,” which is wrong in both cases. Quantitative values are both (50% and 45%, respectively) just below the threshold to malignancy (at 53%) leading to correct assignment for FNH and wrong for HCC. On I_Df_, visual assessment of FNH is “probable benign” and of HCC “probably malignant,” which is correct in both cases. Quantitative values (38% and 55%, respectively) were below and above threshold (50%), respectively, leading also to correct assignments. The metastasis with central necrosis (**c**) shows only a small margin of vital tumour, which can be easier captured by visible judgment than by ROI analysis with unclear tissue boundary. The HCC (**d**) shows the typical behaviour of a haemangioma (see Fig. 1a) and the assignment is false negative. However, the hypointense area of the left liver lobe adjacent to the lesion on *b*-800 indicates motion influences, which raise the diffusion coefficient of the liver tissue and lesion artificially as can be seen on I_D_, I_ADC_, and I_Df_ index maps. *CCC* Cholangiocellular carcinoma, *FNH* Focal nodular hyperplasia, *HCC* Hepatocellular carcinoma

Examples of DWI and two-colour index maps are given in Figs. 1 and 2.

### Verification of voxel-wise cutoff value applicability

ROC analysis of ROI-based analysed index maps and related original parameter maps revealed similar AUC values (Table 3). The comparison of AUC values revealed no significant differences, as expected, neither between I_ADC_ and ADC in discriminating benign from malignant lesions (0.958 *versus* 0.945, *p* = 0.196), nor between I_D_ and D′ in discriminating haemangiomas from all other lesions (0.985 *versus* 0.985, *p* = 1.000), nor between I_f_ and f′ in discriminating FNHs and all other lesions (0.968 *versus* 0.974, *p* = 0.294).

**Table. 3:** Receiver operating characteristic analysis for construction of index maps

Parameter	Mean ± SD	Mean ± SD	*p* value	Dir	AUC	95% Confidence interval	Cutoff	Sen	Spec	Acc
	Malignant (*n* = 74)	Benign (*n* =35)								
ADC	1,124 ± 180	1,692 ± 313	< 0.001	>	0.958	0.922−0.993	1,338.5	0.892	0.914	0.899
I_ADC_	80 ± 21	17 ± 25	< 0.001	<	0.945	0.894−0.996	53.4	0.865	0.914	0.881
	All other (*n* = 86)	HAEMs (*n* = 23)								
D′	1,076 ± 184	1,784 ± 314	< 0.001	>	0.985	0.965−1.000	1,529.4	0.988	0.913	0.972
I_D_	95 ± 12	20 ± 25	< 0.001	<	0.985	0.966−1.000	51.0	0.988	0.913	0.972
	All other (*n* = 97)	FNHs (*n* = 12)								
f′	63 ± 35	164 ± 58	< 0.001	>	0.968	0.938−0.998	114.5	0.907	1.000	0.917
I_f_	82 ± 17	32 ± 17	< 0.001	<	0.974	0.947−1.000	54.6	0.918	1.000	0.927

Mean values and standard deviations of apparent diffusion coefficient (ADC), estimated diffusion coefficient (D′), estimated perfusion fraction (f′), and index maps I_ADC_, I_D_, and I_f_ are presented. The optimal cutoff point of ROC analysis was selected according to maximum Youden index. ADC and D′ values are given in units of 10^-6^ mm^2^/s, f′ values are given in units of 10^-3^, and I_ADC_, I_D_, and I_f_ are given as percentages. *Acc* Accuracy, *ADC* Apparent diffusion coefficient, *AUC* Area under the curve, *Dir* Test direction (">"/"<" means that a lower/higher test result indicates a more positive test), *FNH* Focal nodular hyperplasias, *HAEM* Haemangioma, *SD* Standard deviation, *Sens* Sensitivity, *Spec* Specificity

### Quantitative evaluation of index maps I_ADC_ and I_Df_

All parameter values were significantly lower for malignant lesions than for benign (Table 4), *e.g.*, for ADC 1,124 ± 180 × 10^-6^ mm^2^/s (mean ± standard deviation) *versus* 1,692 ± 313 × 10^-6^ mm^2^/s (*p* < 0.001). Accordingly, for all index values, the numbers of red voxels were significantly higher for malignant than for benign lesions, *e.g.*, for I_ADC_ 80% ± 21% *versus* 17% ± 25% (*p* < 0.001) and for I_Df_ 76% ± 17% *versus* 20% ± 18% (*p* < 0.001).

**Table. 4:** Receiver operating characteristic analysis for differentiation of malignant from benign liver lesions

Parameter	Malignant (*n* = 74) Mean ± SD	Benign (*n* = 35) Mean ± SD	*p* value	Dir	AUC	95% Confidence interval	Cutoff	Sen	Spec	Acc
ADC	1,124 ± 180	1,692 ± 313	< 0.001	>	0.958	0.922−0.993	1,338.5	0.892	0.914	0.899
D′	1,057 ± 188	1,580 ± 387	< 0.001	>	0.902	0.842−0.962	1,173.6	0.757	0.886	0.798
f′	63 ± 31	97 ± 70	0.010	>	0.622	0.491−0.754	114.5	0.932	0.457	0.780
I_ADC_	80 ± 21	17 ± 25	< 0.001	<	0.945	0.894−0.996	53.4	0.865	0.914	0.881
I_D_	94 ± 12	47 ± 43	< 0.001	<	0.782	0.672−0.891	51.0	0.986	0.600	0.862
I_f_	81 ± 16	65 ± 30	0.032	<	0.627	0.499−0.756	57.0	0.905	0.457	0.761
I_Df_	76 ± 17	20 ± 18	< 0.001	<	0.975	0.950−1.000	50.2	0.932	0.943	0.936

Mean values and standard deviations of apparent diffusion coefficient (ADC), estimated diffusion coefficient (D′), estimated perfusion fraction (f′), and index maps I_ADC_, I_D_, I_f_ and combined I_Df_ are presented. The optimal cutoff point of ROC analysis was selected according to maximum Youden index. ADC and D′ values are given in units of 10^-6^ mm^2^/s, f′ values are given in units of 10^-3^, and I_ADC_, I_D_, I_f_, and I_Df_ are given as percentages. *Acc* Accuracy, *ADC* Apparent diffusion coefficient, *AUC* Area under the curve, *Dir* Test direction (">"/"<" means that a lower/higher test result indicates a more positive test), *SD* Standard deviation, *Sens* Sensitivity, *Spec* Specificity

As can be seen in Table 4, among the single parameters ADC, D’, and f′, the ADC was best suited to discriminate benign and malignant lesions. The AUC value of ADC was significantly higher than that of D′ (0.958 *versus* 0.902, *p* = 0.001) and f′ (0.958 *versus* 0.622, *p* < 0.001), AUC of D′ was significantly higher than that of f’ (0.902 *versus* 0.622, *p* = 0.001). By ADC, 89.9% of the lesions were correctly identified as malignant and benign (cutoff value 1,338.5 × 10^-6^ mm^2^/s). By using the combination of D′ and f′, 93.6% of the lesions were correctly identified (cutoff values 1,529.4 × 10^-6^ mm^2^/s and 114.4 × 10^-3^, respectively), which was an improvement compared to ADC.

Comparing the AUC values of I_Df_ and I_ADC_, larger values were found for I_Df_ than for I_ADC_ (0.975 *versus* 0.945), but differences were not significant (*p* = 0.168). The diagnostic accuracy was higher for I_Df_ than for I_ADC_. With I_Df_ 93.6% of the lesions (cutoff value 50.2%) were correctly identified as benign and malignant, with I_ADC_ 88.1% (cutoff value 53.4%). Falsely identified cases by I_Df_ *versus* I_ADC_ were 1 *versus* 2 FNHs, 1 *versus* 1 haemangiomas, 4 *versus* 3 HCCs, 0 *versus* 1 CCCs, and 1 *versus* 6 metastases. I_Df_ was superior to I_ADC_ especially in case of metastases identifying 5 cases correctly as malignant, which were falsely assigned as benign by I_ADC_.

### Visual evaluation of index maps I_ADC_ and I_Df_

By visual judgment of I_Df_ and I_ADC_ maps within translucent hyperintensity from DWI *b*-800 images (Table 5), a similar number of lesions were correctly identified as by quantitative analysis using ROIs excluding central necrosis, cystic components, and scars (95.4% instead of 93.6% for I_Df_ and 90.8% instead of 88.1% for I_ADC_). As in the quantitative analysis, the reached diagnostic accuracy was higher for I_Df_ than for I_ADC_. With I_Df_ 95.4% of the lesions were correctly identified, with I_ADC_ 90.8%. The assignment was “definite” in 87.2% for I_Df_ and in 89.9% for I_ADC_ and “probable” in 12.8% for I_Df_ and in 10.1% for I_ADC_. “Probable” assignment by I_Df_ and I_ADC_ was mainly found for FNHs (4 and 7, respectively) and HCCs (4 and 6, respectively) and only rarely for haemangiomas (0 and 1, respectively), CCCs (1 and 0, respectively), and metastases (2 and 0, respectively). Falsely identified cases by I_Df_ *versus* I_ADC_ were 2 *versus* 5 FNHs, 1 *versus* 1 haemangiomas, 1 *versus* 3 HCCs, 0 *versus* 0 CCCs, and 1 *versus* 1 metastasis. I_Df_ was superior to I_ADC_ especially in case of FNHs and HCCs identifying 3 FNHs and 2 HCCs correctly, which were falsely assigned by I_ADC_. Examples are given in Fig. 1b, Fig. 2a, and Fig. 2b. Visual judgment of I_Df_ was superior especially in case of HCCs identifying 4 HCCs correctly, which were falsely assigned by quantitative analysis. Visual judgment of I_ADC_ was superior especially in case of metastases but inferior in case of FNHs identifying 5 metastases correctly, which were falsely assigned by quantitative analysis, and 3 FNHs falsely (as “probable malignant”), which were correctly identified by quantitative analysis.

**Table. 5:** Results of visual judgment of index maps I_ADC_, I_D_, I_f_, and combined I_Df_

Parameter	Definite				Probable				All					
	TN	FN	FP	TP	TN	FN	FP	TP	N	P	T	Sen	Spec	Acc
Investigator 1														
I_ADC_	28	2	3	65	1	2	3	5	35	74	109	0.946	0.829	0.908
I_D_	22	1	13	73	0	0	0	0	35	74	109	0.986	0.629	0.872
I_f_	6	1	23	73	4	0	2	0	35	74	109	0.986	0.286	0.761
I_Df_	26	2	1	66	6	0	2	6	35	74	109	0.973	0.914	0.954
Investigator 1 repeated after 4 months														
I_ADC_	28	2	3	65	1	2	3	5	35	74	109	0.946	0.829	0.908
I_Df_	26	2	1	66	6	0	2	6	35	74	109	0.973	0.914	0.954
Investigator 2														
I_ADC_	28	2	3	65	1	2	3	5	35	74	109	0.946	0.829	0.908
I_Df_	26	2	1	66	6	0	2	6	35	74	109	0.973	0.914	0.954

*Acc* Accuracy, *FN* False negative cases, *FP* False positive cases, *N* Number of benign cases (TN + FP), *P* Number of malignant cases (TN + FP), *T* Total number of cases (N + P), *TN* True negative cases, *Sen* Sensitivity, *Spec* Specificity, *TP* True positive cases

The repeated analysis by the same investigator and by the independent investigator (see Table 5) revealed excellent intraobserver and interobserver reliability (ICC_intra_ 0.992 for I_ADC_ and 0.989 for I_Df_; ICC_inter_ 0.986 for I_ADC_ and 0.977 for I_Df_).

## Discussion

In this study, simplified IVIM was used to create combined two-colour index maps I_Df_ from parameters D′ and f′ as overlay to *b*-800 images in order to facilitate visual assessment of liver lesions. Red voxels show diffusion and perfusion restrictions and indicate malignancy in combination with translucent *b*-800 hyperintensity. The main result was that the voxel-wise combination of D′ and f′ thresholds in the form of the I_Df_ index map provides identical diagnostic accuracy as the ROI-based combined analysis of the D′ and f′ parameter maps. A higher diagnostic accuracy was found for I_Df_ than for I_ADC_ (created from ADC). Visual judgment of the I_Df_ index map as two-colour overlay to *b*-800 images showed comparable diagnostic accuracy than quantitative analysis of I_Df_.

In previous simplified IVIM studies on liver lesions at 1.5 and 3.0 T it was found that ADC is the best single parameter to discriminate between malignant and benign liver lesions but that improved discriminatory power could be reached by combined use of D′ and f′ [14, 15]. This result was confirmed in the present study. Compared to the previous 1.5-T study [15], which was performed on the same patient group than the present study but with new ROI analysis, higher diagnostic accuracy was reached, for ADC (89.9% *versus* 82.1%) and for combined D′ and f′ (93.6% *versus* 85.6%). In the present study, one reference ROI per lesion type and patient was included, in the previous study up to 5 lesions per lesion type and patient were included and averaged for analysis (clustered analysis). Necrotic areas, liquids, and scares were excluded from ROIs in both studies, but can also be excluded retrospectively by automatically selecting voxels with low diffusion coefficients with the help of histogram analysis of D′ (upcoming study by Mesropyan et al).

New in the present work is the creation and evaluation of the index maps I_Df_, which combine the information from D′ and f′, use only two colours, and are presented as overlay to *b*-800 in order to be able to assess only the vital tumour areas by translucent hyperintensity and to exclude necrosis, cystic components, and scars from assessment. Up to now, colour-coded maps with more than two colours have been used for the different IVIM parameters [13, 21, 22, 32], sometimes presented as overlays to b0 images [14, 15]. Whether the ROI-wise obtained and combined cutoff values of D′ and f′ would also work voxel-wise in I_Df_ was not clear in advance. Perfusion and diffusion restrictions do not necessarily have to occur in the same voxels. But the fact that I_Df_ provided identical diagnostic accuracy than combined use of D′ and f′ (93.6% *versus* 93.6%) means that the ROI-wise obtained cutoff values of the parameters can be applied voxel-wise in the index maps.

For I_Df_ higher accuracy was reached than for I_ADC_, by quantitative analysis (93.6% *versus* 88.1%) and by visual judgment (95.4% *versus* 90.8%). The relative good performance of I_ADC_ is due to the fact that for liver lesion differentiation diffusion and perfusion influences act in the same direction.

When visually assessing two-colour index maps, it is only necessary to distinguish whether more or less than half of the voxels in the tumour areas of interest are red. This allows a rapid and easy image interpretation also for less skilled operators. Excellent intraobserver and interobserver reliability was achieved. By visual judgment comparable diagnostic accuracy was reached than by ROI-based quantitative analysis, for I_Df_ (95.4% *versus* 93.6%) and I_ADC_ (90.8% *versus* 88.1%). The assignment malignant/benign was “definite” in about 90% of the cases and “probable” in about 10%, for I_Df_ and I_ADC_. Some of the FNHs showed relatively high numbers of red voxels on I_Df_ with scattered distribution caused by heterogeneous perfusion as can be seen on I_f_ index map. Those FNHs looked similar to typical HCCs (Fig. 2a, b). Visual assessment of those lesions was less accurate than ROI-based quantitative analysis. Metastases, on the other hand, often have only a narrow margin of vital tumour tissue, so that an exact ROI positioning is difficult leading to less accurate results in case of quantitative analysis compared to visual assessment (Fig. 2c).

General concerns regarding the simplified IVIM approach as for example the b value choice have already been addressed in the previous studies [14, 15]. Only three of the four acquired b values were used, because no diagnostic added value was found for the fourth *b* value (250 s/mm^2^) and the determination of D* [14, 15]. Simplified IVIM parameter calculations by using approximations and explicit formulas instead of fitting procedures are simple and stable and lead to reliable information. Exceptions are as generally low signal-to-noise ratios (*e.g.*, patients with hemochromatosis or fatty liver), small lesions (partial volume effects), or presence of artifacts. Due to motion influences artificially enlarged D′ and reduced f′ values may be measured, especially for the left liver lobe and on slices close to the heart. It is important to check the surrounding liver in the *b*-800 image for signal loss (Fig. 2d). Since the IVIM parameter f depends on the relaxation times, f may vary with field strength and sequence parameters used (especially b values, echo times, and repetition times) [33, 34], this also applies to the cutoff points used for the index maps. The new combined two-colour index maps I_Df_ were checked on the same patients who provided the cutoff points for generation in order to enable a direct comparison with the combined use of the parameter maps D′ and f′. A validation study is planned on a larger patient cohort, which also includes rarer and atypical lesions as well as lesions difficult to identify in DWI. It is interesting to compare the use of the two-colour index maps I_Df_ with full set of conventional protocol in terms of reading time and reader confidence.

In conclusion, the voxel-wise combined index maps I_Df_ and the ROI-based combination of D′ and f′ parameters provide concordant diagnostic accuracy for the differentiation of malignant and benign liver lesions. The I_Df_ index map used as two-colour overlay to *b*-800 images can be considered as a new tool for visual assessment of liver lesion malignancy.

## Data Availability

The datasets used and/or analysed during the current study are available from the corresponding author on reasonable request.

## Abbreviations

ADC: Apparent diffusion coefficient
AUC: Area under the curve
CCC: Cholangiocellular carcinoma
CRC: Metastasis from colorectal carcinoma
D′: Estimated diffusion coefficient
DWI: Diffusion-weighted imaging
f′: Estimated perfusion fraction
FNH: Focal nodular hyperplasia
HCC: Hepatocellular carcinoma
I_ADC_: ADC index maps
ICC: Intraclass correlation coefficient
I_D_: D′ index maps
I_Df_: D′ and f′ combined index maps
I_f_: f′ index maps
IVIM: Intravoxel incoherent motion
MRI: Magnetic resonance imaging
ROC: Receiver operating characteristic
ROI: Region of interest
